# An analysis of factors affecting the mercury content in the human femoral bone

**DOI:** 10.1007/s11356-016-7784-9

**Published:** 2016-10-13

**Authors:** A. Zioła-Frankowska, M. Dąbrowski, Ł. Kubaszewski, P. Rogala, A. Kowalski, M. Frankowski

**Affiliations:** 1 Faculty of Chemistry, Department of Analytical Chemistry, Adam Mickiewicz University in Poznań, Umultowska 89b, 61-614 Poznan, Poland; 2 Department of Spondyloorthopaedics and Biomechanics of the Spine, W. Dega University Hospital, Poznan University of Medical Sciences, 28 Czerwca 1956 135/147, 61-545 Poznan, Poland; 3 Department of Orthopedic and Traumatology, W. Dega University Hospital, Poznan University of Medical Sciences, 28 Czerwca 1956 135/147, 61-545 Poznan, Poland; 4 Faculty of Chemistry, Department of Water and Soil Analysis, Adam Mickiewicz University in Poznań, Umultowska 89b, 61-614 Poznan, Poland

**Keywords:** Mercury, Femoral bone, CV-AFS technique, Environment factors, Body mass index

## Abstract

The study was carried out to determine the content of mercury in bone tissue of the proximal femur (head and neck bone) of 95 patients undergoing total hip replacement due to osteoarthritis, using CF-AFS analytical technique. Furthermore, the investigations were aimed at assessing the impact of selected factors, such as age, gender, tobacco smoking, alcohol consumption, exposure to chemical substance at work, type of degenerative changes, clinical evaluation and radiological parameters, type of medications, on the concentration of mercury in the head and neck of the femur, resected in situ. Mercury was obtained in all samples of the head and neck of the femur (*n* = 190) in patients aged 25–91 years. The mean content of mercury for the whole group of patients was as follows: 37.1 ± 35.0 ng/g for the femoral neck and 24.2 ± 19.5 ng/g for the femoral head. The highest Hg contents were found in femoral neck samples, both in women and men, and they amounted to 169.6 and 176.5 ng/g, respectively. The research showed that the mercury content of bones can be associated with body mass index, differences in body anatomy, and gender. The uses of statistical analysis gave the possibility to define the influence of factors on mercury content in human femoral bones.

## Introduction

It is well known that mercury is an element which is highly toxic for humans, animals, plants, and microorganisms (Wolfe et al. 1998; Wang et al. 2012, Pérez-Sanz et al. 2012, Kowalski and Frankowski 2015, Sanchez-Chardi et al. 2007). It exhibits mutagenic and teratogenic properties, and can be accumulated in the human body (Clarkson et al. 2007, Kowalski and Frankowski 2015). Mercury exists in several forms, as inorganic mercury (metallic mercury and mercury vapor—Hg^0^; mercurous—Hg_2_^++^; or mercuric—Hg^2+^ salts) and organic mercury (compounds with methyl, ethyl, phenyl, or similar groups) (Syversen and Kaur 2012). Methylmercury (Me-Hg) is the most toxic form, Hg^2+^ and Hg^+1^ compounds are less toxic, and Hg^0^ is the least toxic form (Bernhoft 2012). Mercury is mainly used in the production of biocides, batteries, and dental amalgam fillings in industrial electrolysis and in the extraction of gold. However, most of this metal (in the form of Hg^0^ vapor) is emitted into the atmosphere via fossil fuel-based production of electricity (coal, lignite, and crude oil burning) (Lanocha et al. 2013). The toxicity of mercury varies depending on its form, dose, and rate of exposure (Nordberg et al. 2014). For example, elemental mercury is very mobile, mainly due to its volatility; it can easily cross the blood–brain barrier; however, it is quickly oxidized to inorganic mercury in the blood and other tissues (Halbach and Clarkson 1978; Eide and Syversen 1983). The target organ for inhaled mercury vapor is primarily the brain (Nordberg et al. 2014). Mercurous and mercuric salts were found mainly to damage the gut lining and kidneys, while methylmercury can be widely distributed throughout the body. As stated before, mercury toxicity also varies with dosage: a large acute exposure to elemental mercury vapor induces severe pneumonitis, which in extreme cases, can be fatal, while low-grade chronic exposures to elemental or other forms of mercury induce more subtle symptoms and clinical findings (Tchounwou et al. 2003). Contents of Hg in mammalian tissues vary between 0.02 and 0.25 mg/kg, being the highest in kidneys and the lowest in muscles (Jørgensen 2000). Mercury mean concentrations in human fluids were reported at 5.3 μg/l in blood, 2.1 μg/l in serum, and 3.5 μg/l in urine (Reimann and Caritat 1998). Mercury can also be deposited in skeletal elements (Zaichick et al. 2011), where it remains until bone remodeling is complete or the resorption occurs (Miculescu et al. 2011). It should also be pointed out that almost 75 % of heavy metals, including mercury, is deposited in the bones during adolescence, and the value can increase to 95 % in adults who are professionally exposed to metals (Miculescu et al. 2011). Due to the clear influence of mercury on human health, it is necessary to continue investigations on this metal in different human tissues and on the possible health effects posed by particular mercury concentrations.

The primary aim of this study was to mark using cold vapor atomic fluorescence spectrometry (CV-AFS) method the content of mercury in the femoral head (cancellous bone) and femoral neck (cortical bone) of patients subjected to total hip replacement surgery for osteoarthritis. The current study was performed to (1) find differences in the content of mercury between the femoral head and neck, according to gender, age and body mass index (BMI); (2) estimate possible correlations between the content of mercury in the femoral bone and selected factors; and (3) investigate whether the determined content of mercury in the femoral head and neck may be indicative of increased risk.

## Materials and methods

### Bone samples

The samples from 95 patients were taken during surgical operation on for total hip replacement (THR). The study was approved by the Bioethics Committee of the Karol Marcinkowski University of Medical Sciences in Poznań, Poland (approval no. 172/14). Patients included in the study gave their consent to use their tissues for research purpose and made the personal query. The femoral bone was obtained intraoperatively from the patients, acquired during hip replacement procedure. In all cases, the indication for the procedure was idiopathic osteoarthoris of the hip joint. The analysis was executed for two anatomic regions of each type of resected fragment in situ with an orthopedic oscillating saw: the femoral head (FH) and femoral neck (FN). The samples were taken without articular capsule and without articular cartilage. After resection, the sample was frozen in −20 °C.

In the study, female patients were included in six age groups (A 20–40; B 41–50; C 51–60; D 61–70; E 71–80; F >80) and male patients in five age groups; there were no male patients from age group A (20–40). Detailed and accurate information about characteristics of patients and femoral bone samples are presented in the studies by Zioła-Frankowska et al. (2015a, b).

### Analytical methods

The frozen femoral bone samples were freeze dried using a Lyovac lyophilizer GT2e (Steris, Germany) for 24 h. After drying, the samples were weighed and mineralized with suprapure nitric acid (V) (Merck, Germany) in a microwave system (Mars 5 Xpress CEM, USA) according to procedure developed by, e.g., Zioła-Frankowska et al. (2015a). Mercury concentrations were determined (in the three replications) using a CV-AFS (Millennium Merlin Analyzer 10.025, PS Analytical, England) in all tested femoral bone samples. The percent relative standard deviation (% RSD) for the CV-AFS analytical technique did not exceed 5 %. The analytical agents used for mercury determination were of the highest available analytical grade. For the preparation of the reducing solution, the Tin (II) chloride was used (Merck, Darmstadt, Germany). The working standard solutions were prepared on the day of analysis by diluting the stock standard solution of Hg(NO_3_)_2_ (1000 ± 4 mg L^−1^) with 12 % HNO_3_ (Sigma-Aldrich, USA). The laboratory glass used were made of boron–silica glass of the highest quality and were soaked in 10 % (*v*/*v*) nitric acid (Sigma-Aldrich, USA) for 24 h and washed three times with deionized water (water was previously purged with argon for 6 h to remove traces of mercury). Additionally, to check and ensure the applied method, a standard reference material SRM 1515 Apple Leaves (National Institute of Standards and Technology, USA) was used. The SRM which is suitable for human bone (SRM 1400 of bone ash from National Institute of Standard and Technology) is not certified for mercury which is not present in composition of SRM material. The method recovery was 98.1 ± 3.5 %. The limit of quantification (LOQ) of the analytical instrument was 0.8 ng Hg L^−1^. Presented analytical method for the determination of total mercury has been applied by, e.g., Kowalski and Frankowski (2015, 2016) and Siudek et al. (2016). Based on obtained data of mercury content in femoral head and neck samples, the statistical analysis was conducted with PL v.7.0 (StatSoft, USA) software. The Shapiro–Wilk and Mann–Whitney *U* tests (*p* < 0.05) were used to check the normal distribution of results and to investigate significant differences and relationship between environmental factors and mercury content, respectively.

## Results

Mercury content was significantly higher in the femoral neck (Table 1). The results of mercury content in samples of the femoral head and neck samples according to different factors are presented in Table 1.

**Table 1:** Content of mercury (in ng/g on dry mass basis) in the femoral head and neck samples, according to various factors (*N* = 95)

Factors		Femoral head			Femoral neck		
		Med. Q1–Q3 (ng/g)	Min–max (ng/g)	M–WU	Med. Q1–Q3 (ng/g)	Min–max (ng/g)	M–WU
Total mercury content		17.3 (11.7–32.2)	(3.6–128.5)	0.01	26.3 (12.5–48.5)	(2–176.5)	0.01
Age	<60 (*n* = 37)	15.2 (11.7–29.5)	(7.4–90.2)	NS	24.9 (12.2–39.3)	(8–146)	NS
	>60 (*n* = 58)	18 (11.8–32.8)	(3.6–128.5)		26.85 (13–50.8)	(2–176.5)	
Gender	Women (*n* = 57)	15.7 (10.3–27.6)	(3.6–128.5)	NS	25.1 (12.2–44.2)	(2–169.6)	NS
	Men (*n* = 38)	20.05 (13.1–32.7)	(8.4–90.2)		30.7 (14.7–48.6)	(5.9–176.5)	
Type of osteoarthritis	Primary *idiopathic* (*n* = 54)	18 (11.8–32.7)	(3.6–65.7)	NS	29.8 (13.1–48.5)	(2–169.6)	NS
	Secondary developmental dysplasia of the hip (*n* = 41)	15.2 (11.3–28.5)	(7.4–128.5)		23.8 (11.8–40)	(6.7–176.5)	
Avascular necrosis of the femoral head	Yes (*n* = 13)	20.2 (14.3–38.6)	(8.8–90.2)	NS	23.5 (16.4–51.9)	(6.7–176.5)	NS
	No (*n* = 82)	15.8 (11.3–31)	(3.6–128.5)		26.5 (12.2–40)	(2–169.6)	
Place of residence	City (*n* = 71)	15.5 (11.7–32.2)	(7.2–90.2)	NS	26.3 (12.5–44.2)	(5.9–169.6)	NS
	Village (*n* = 24)	20.05 ( 11.6–28.95)	(3.6–128.5)		27.6 (13.5–52.85)	(2–176.5)	
Contact with paints	No (*n* = 86)	16.6 (11.7–29.5)	(3.6–128.5)	NS	27.65 (13.3–50.8)	(2–176.5)	<0.01
	Yes (*n* = 9)	32.7 (11.8–38.6)	(8.4–90.2)		11.8 (9.4–16.4)	(5.9–31.3)	
Chemicals	No (*n* = 66)	17.3 (11.7–31)	(3.6–128.5)	NS	26.7 (14.8–48.5)	(2–176.5)	NS
	Yes (*n* = 29)	15 (11.8–32.7)	(8.4–90.2)		16.4 (10–40)	(5.9–99.3)	
Physical activity	Yes (*n* = 80)	17.3 (11.9–31.9)	(3.6–128.5)	NS	26.9 (13.2–48.6)	(2–176.5)	NS
	No (*n* = 15)	14.3 (9.7–32.2)	(8.3–90.2)		15.6 (11–34.4)	(6.7–169.6)	
Fish consumption	No (*n* = 87)	17.3 (11.7–32.2)	(3.6–128.5)	NS	26.3 (13.1–48.5)	(2–176.5)	NS
	Yes (*n* = 8)	14.3 (11.2–25.25)	(7.2–37.6)		22 (11.4–42.9)	(9.7–51.5)	
High tea consumption	Yes (*n* = 16)	22.75 (13–32.5)	(9.7–90.2)	NS	30.4 (14–60.9)	(8.9–146)	NS
	No (*n* = 79)	15.9 (11.1–31)	(3.6–128.5)		25.7 (12.2–44.2)	(2–176.5)	
High coffee consumption	Yes (*n* = 9)	19.9 (11.8–28.2)	(9.4–65.7)	NS	55.1 (24.9–80.7)	(8.9–176.5)	NS
	No (*n* = 86)	15.8 (11.7–32.2)	(3.6–128.5)		25.9 (12.5–39.3)	(2–169.6)	
Smoking	Non-smoker (*n* = 72)	16.6 (11.9–28.3)	(3.6–128.5)	NS	26.5 (13.8–48.55)	(2–176.5)	NS
	Smoker (*n* = 23)	23.3 (11.1–34.2)	(7.9–90.2)		24.9 (10.4–44.2)	(8.1–101)	
Alcohol consumption	Yes (*n* = 49)	18.3 (12–32.7)	(7.9–90.2)	NS	30.4 (12.5–51.9)	(5.9–176.5)	NS
	No (*n* = 46)	15.6 (11.1–28.2)	(3.6–128.5)		25.35 (13–37.4)	(2–169.6)	

*Med.* median, *QL* lower quartile, *QU* upper quartile, *M–WU* Mann–Whitney *U* test, *p* level of significance, *NS* difference not significant

**Fig. 1:**
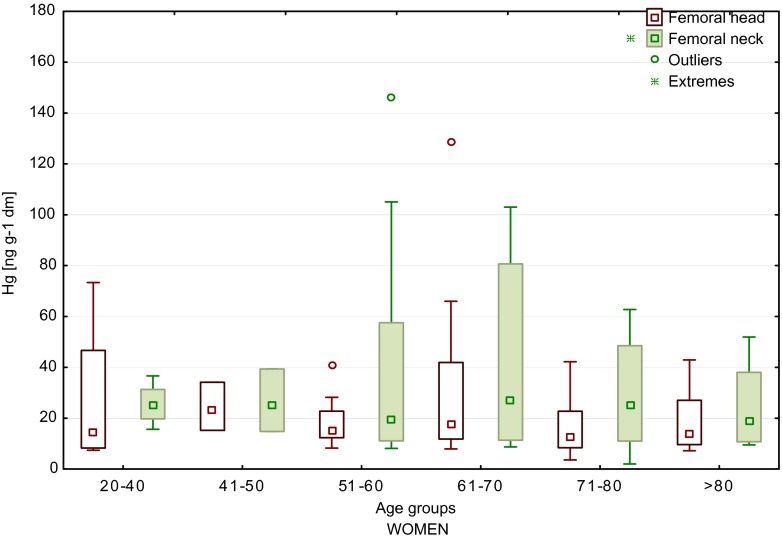
Median values of mercury content of the femoral head and neck of women, depending on age (*boxes* indicate upper and lower quartile, *whiskers* indicate minimum and maximum values, *open circles* indicate outlier values, and *asterisks* indicate extreme values). No significant difference between the groups

For women, the highest content of mercury in the neck of the femur (169.6 ng/g) was detected in the sample from age group E and the highest value in the femoral head (128.5 ng/g) for a sample from age group D. In the femoral neck samples, we observed higher content of mercury as compared to the femoral head (Fig. 1). Also, for a sample from age group 71–80, we found that mercury content was 20 times lower in the femoral head (8.4 ng/g in FH and 169.6 ng/g in FN, age 79). As well, the content of mercury increased with age, particularly for femoral neck (FN) samples. In female patients younger than 40 years old, the average mercury content in FN samples was 24.3 ng/g and in patients >40–37.2 ng/g.

**Fig. 2:**
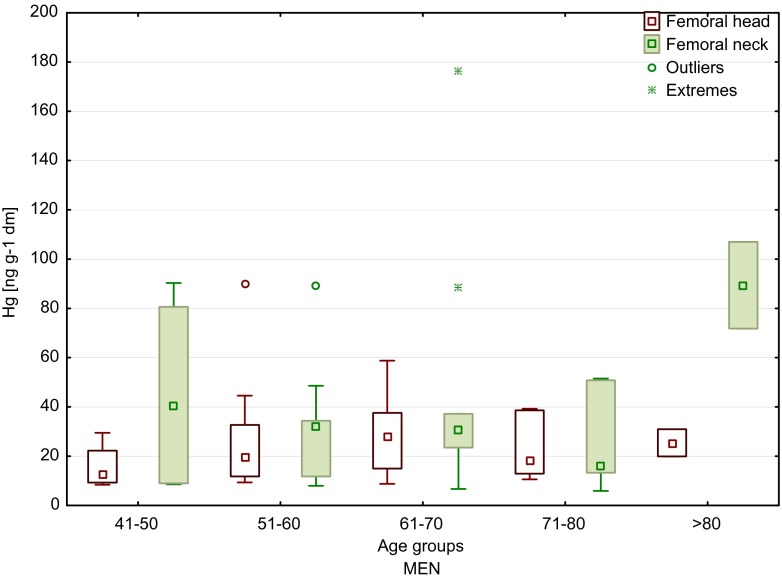
Median values of mercury content of the femoral head and neck of men, depending on age (*boxes* indicate upper and lower quartile, *whiskers* indicate minimum and maximum values, *open circles* indicate outlier values, and *asterisks* indicate extreme values). No significant difference between the groups

The lowest value for male femoral head samples was observed in age group B (8.4 ng/g) and the highest in age group C (90.2 ng/g). While for femoral neck samples, the lowest content was obtained in age group E (5.9 ng/g) and the highest in age group D (176.5 ng/g). In terms of age of male patients, it was found that the highest content of mercury occurred in the groups of younger patients (<60 years of age) and the oldest patients (median 40.2 and 89.4 ng/g, respectively). However, these age groups were not numerous (*n* = 4 and *n* = 2, respectively), and the result can be overestimated. Moreover, considering all male patients, it was found that mercury contents were higher in femoral neck than in femoral head samples (similar trend as in the case of female samples) (Fig. 2).

**Fig. 3:**
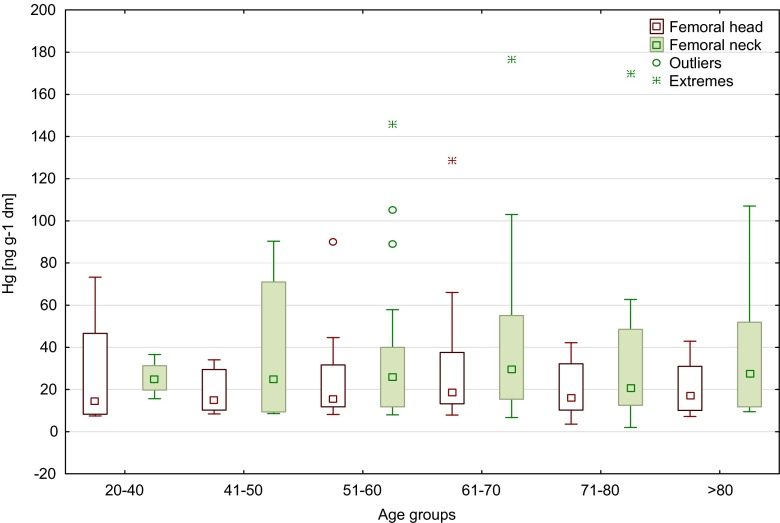
Median values of mercury content of the femoral head and neck of all patients, depending on age (*boxes* indicate upper and lower quartile, *whiskers* indicate minimum and maximum values, *open circles* indicate outlier values, and *asterisks* indicate extreme values)

The content of mercury in the femoral head and neck of men and women, depending on age, is shown in Fig. 3. There was no significant differences observed between age groups <60 and >60 (Table 1). The largest age group of all patients was 61–70 years old, and the highest mercury content was determined in that age range (means 29.6 ng/g in FH and 42.7 ng/g in FN). Comparing the remaining age groups revealed the highest level of mercury in the femoral neck in groups 51–60 and 61–70 years (median 32.1 and 30.4 ng/g, respectively) and in the femoral head in the age group 61–70 years (median 28.2 ng/g) (Fig. 3). In all 95 patients, we did not notice any significant differences between age groups in women and men for femoral neck and head (Kruskal–Wallis and Mann–Whitney *U* test). However, we observed a tendency to decrease mercury content with age in the neck of the femur in men (median from 40.2 ng/g in age group 41–50 to 16.4 ng/g in age group 71–80).

**Fig. 4:**
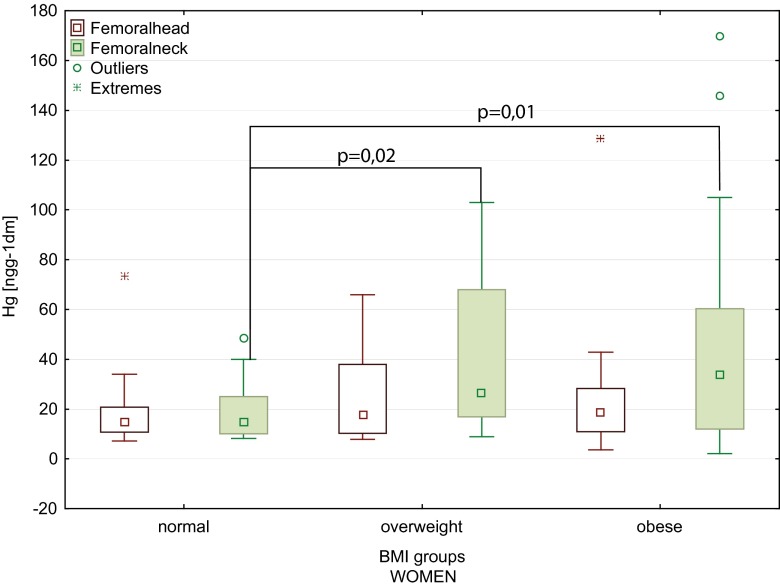
Mercury content of the femoral head and neck of women, depending on the BMI (*boxes* indicate upper and lower quartile, *whiskers* indicate minimum and maximum values, *open circles* indicate outlier values, and *asterisks* indicate extreme values). Normal BMI, between 18.5 and 24.9; overweight, between 25 and 29.9; obese, −30 and above)

**Fig. 5:**
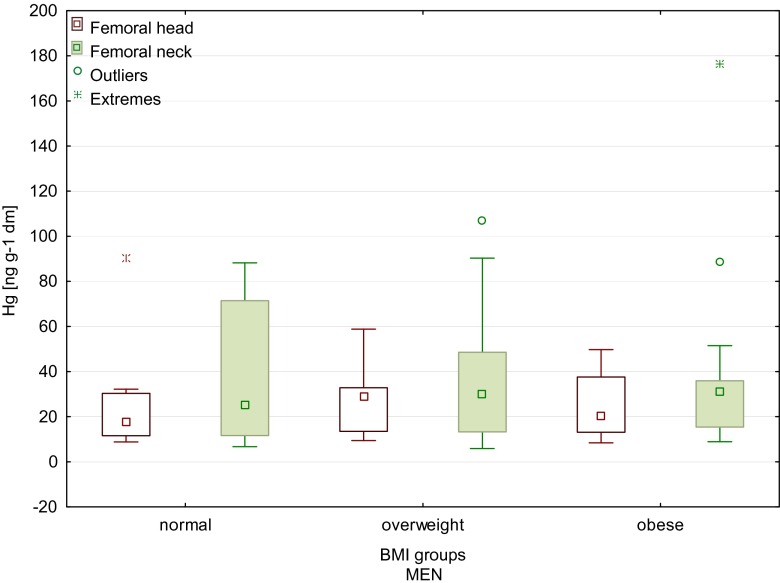
Mercury content of the femoral head and neck of men, depending on the BMI (*boxes* indicate upper and lower quartile, *whiskers* indicate minimum and maximum values, *open circles* indicate outlier values, and *asterisks* indicate extreme values). No significant difference between the groups. Normal BMI, between 18.5 and 24.9; overweight, between 25 and 29.9; obese, −30 and above)

Furthermore, the statistically significant increase in the mercury content of the femoral head and neck that correlated with the increase in body weight was observed (Table 2). Also, higher values of the body mass index (BMI) were conducive to higher accumulation of mercury in the femoral neck for all patients (Table 2). A statistically significant increase in the mercury content of the femoral neck in women with the increase in body weight and BMI (0.37 and 0.37, respectively) was found. Figure 4 shows higher mercury content of the femoral head and neck in overweight and obese female patients. We showed statistically significant difference in Hg content between a normal BMI group and overweight group (Mann–Whitney *U* test *p* = 0.02). Similar difference was found between a normal BMI group and obese group (Mann–Whitney *U* test: *p* = 0.01; Kruskal–Wallis test *p* = 0.016). There were no significant differences between the mercury contents in the femur in men, depending on body weight and BMI (Fig. 5).

**Table 2:** Spearman correlation of the mercury content with clinical factors (*N* = 95)

	Femoral head		Femoral neck	
Clinical factors	*R*	*p* value	*R*	*p* value
Age	−0.03	0.75	0.04	0.73
Body weight	0.21*	0.04*	0.24*	0.02*
Height	0.15	0.16	0.01	0.89
BMI^a^	0.18	0.08	0.28*	0.01*
NRS^b^	0.00	1.00	0.11	0.30
Kellgren–Lawrence^c^	0.07	0.50	−0.04	0.67
Width of cortical bone	0.13	0.21	0.13	0.19
Width of the bone	0.24*	0.02*	0.29*	0.01*
Cortical index	−0.06	0.54	−0.08	0.43

*Statistically significant

^a^Calculation by formula BMI = Weight / (Height / 100)^2^ [kg/m^2^]

^b^Numerical Rating Scale

^c^Kellgren & Lawrence (1957)

Table 2 presents Spearman correlation between mercury content and clinical factors for all patients included in the study. We observed that the severity of osteoarthritis (according to Kellgren–Lawrence scale) and pain intensity (according to visual analog scale [VAS]) was not correlated with mercury content of the femoral head and neck. Other radiological parameters connected with bone sizes (width of cortical bone, cortical index) did not correlate with mercury content (Table 2).

Mercury content was analyzed in the female patients who were taking various types of medications regularly at the time of the study. Among women, the biggest group (*n* = 26) consisted of patients taking cardiacs and medications to regulate blood pressure. It is worthy to underline that the highest content of mercury in femoral neck and head was obtained in this group of female patients. There were no significant differences observed between taking and not taking cardiac medicaments for femoral neck and head (Mann–Whitney *U* test; *p* = 0.42). Despite this, based on the comparison of mean mercury content, we observed the higher content of mercury in the femoral bone from patients taking cardiac medications (femoral head 26.57 ng/g; femoral neck 41.97 ng/g) than in those who were not taking such medicaments (femoral head 19.05 ng/g; femoral neck 29.45 ng/g). In male patients, 13 confirmed regularly taking medicines for heart diseases at the time of the research. There were no significant differences between taking and not taking cardiac medications for femoral neck (Mann–Whitney *U* test *p* = 0.98). However, in femoral head, we confirmed that mercury levels are significantly increased in men taking cardiac medications (Mann–Whitney *U* test: *p* = 0.03). We also observed that higher contents of Hg were determined in femoral bone in patients who were not taking medicines for heart diseases. Nonetheless, it should be underlined that in the group of male patients who were not taking drugs for heart diseases, we had femoral neck and head samples with the highest content of mercury.

The content of mercury was also examined in femoral head (FH) and neck (FN) samples of female patients who were cigarette smokers and alcohol drinkers for the duration of the study. Average mercury content in the group of smokers was 33.58 ng/g for FH and 39.14 ng/g for FN samples. In the group of women who declared alcohol drinking, average mercury content were slightly higher in the neck of the femur (34.78 ng/g), as compared to the average mercury content in the head of the femur (20.07 ng/g). In addition, five of the female patients were both smokers and alcohol consumers, and in this group, we did not find any significant differences in the content of mercury between the head and the neck of the femur. In male patients who were smokers, mercury levels ranged from 8.4 to 90.2 ng/g in FH and from 9.4 to 88.2 ng/g in FN. In patients who affirmed as a alcohol drinkers, we observed similar range of mercury content like in the smokers patients, both in the femoral head and neck samples. In patients who were both smokers and alcohol drinkers, there were no differences in contents of mercury between femoral head and neck samples. We did not find significant differences between all smoking patients and those who were not smokers (Table 1).

In the study, it was examined if the profession related directly or indirectly with chemical substances can influence on mercury content in male femur bones. The highest content of mercury (88.9 ng/g) in the group of male patients (industrial professions) was determined in a FH sample of the patient who was a mechanic. The mean mercury contents in the femoral bones of patients who declared working with chemicals were 20.72 ng/g for FH and 30.77 ng/g for FN, and in male patients who did not have contact with chemical substances at work, the mean mercury contents were reported to be 27.84 ng/g for FH and 42.24 ng/g for FN. There were no significant differences observed between group declared or not working with chemicals (Mann–Whitney *U* test; FN *p* = 0.27 and FH *p* = 0.29). The results obtained in this study do not indicate the impact of profession on the level of mercury in femoral samples of men.

## Discussion

It is becoming increasingly important to assess the risk to bone tissue from exposure to metals of environmental and occupational origin. The processes of bone remodeling are active throughout the lifespan and, therefore, can be an indicator of metal accumulation in bone tissue from long-term chronic exposure. The toxic effects may be revealed after many years of exposure or may appear suddenly (Zioła-Frankowska et al. 2015a).

Mercury was detected in all the examined samples of the head and neck of the femur. Much higher levels of mercury were determined in the femoral neck than in the head of the femur, both in male and female samples. In the studies of Lanocha et al., mercury content was also significantly higher in the femoral neck as compared to the femoral head (median of 2.7 vs 1.8 ng/g dry weight) (Lanocha et al. 2012, 2013). It should be underlined that the results of mercury content determined by Lanocha et al. (2012, 2013) were 10 times lower than the values in our study (Table 1). The differences can be associated with the patients who were included in both studies or with the preparation method. In the study by Lanocha et al. (2012, 2013), bone samples were dried; in our study, bone materials were freeze dried. In comparison, Hg content of the cancellous tissue collected from medieval human bones was up to 40 times higher than that of the cortical tissue (Rasmussen et al. 2013). However, the study was carried out only for a very small group (two skeletons). Rasmussen et al. (2013) suggested that such high mercury content of the cancellous tissue taken from the femur and humerus could be associated with the presence of organic mercury forms which tended to concentrate in the soft tissues intermixed with the cancellous bone. In the present study, we concluded that the differences which we noticed could be related with the type of bone tissues (cancellous bone from the femoral head and cortical bone from the femoral neck) and the site from where the tissue was harvested. The same finding was obtained for the analysis of aluminum in the femoral head and neck (Zioła-Frankowska et al. 2015b). Also, it is known that in the formation of cortical and cancellous bones, individual osteons which are separated by cement lines are involved. Besides cortical bone remodels slower then cancellous bones (Pemmer et al. 2013). Pemmer et al. (2013) observed different distributions of Zn, Pb, and Sr in femoral neck and head samples. Also, they obtained higher accumulation of metals in cement lines than in adjacent mineralized bone matrix, which may indicate a possibly different mechanism of analyzed metals uptake. Furthermore, it was indicated that in bone structural units, the concentration of metals depends on the degree of mineralization. These findings also can indirectly explain the differences in the determined content of mercury in our analyzed femoral bones.

Furthermore, the levels of mercury content were different in male and female samples. Lower values were obtained in women, both in the head and in the neck of the femur. However, these differences were not significant (Table 1). The highest content of mercury for both groups of patients was determined in the neck of the femur. Similarly, in the study by Lanocha et al. (2012), mercury levels in the femoral bone from male and female patients did not differ significantly. In contrast, the analysis of mercury in biopsies of human kidney cortex revealed three times higher concentrations in women than in men (Vahter et al. 2002). Also, it was found that mercury concentrations in human hair samples obtained for women were higher as compared to men (Michalak et al. 2014; Olivero et al. 2002; Agusa et al. 2007).

At the same time, Barbosa et al. (2001) found significantly lower total mercury concentration in women hair samples than in men in their study. The similar observation was found in the research by Skalnaya et al. (2016).

Although there were no significant correlations between mercury content of the femoral bone and the age of the patients who have taken part in the present study, we observed differences in the levels of mercury between the age groups. In women, a significant increase in the concentration of mercury in the neck of the femur was reported for patients >50 years of age (Fig. 1), and in men, a significant increase in mercury content was found in femoral neck samples of the oldest patients (>80 years), as compared to younger ones (41–50 years old) (Fig. 2). Similar in the study by Skalnaya et al. (2016), the content of Hg in hair was increased in men with age. Furthermore, considering all patients (Fig. 3), it was observed that the highest concentrations of mercury, both in the head and in the neck of the femur, were determined in patients >50 years of age. Also the higher content of mercury in blood samples was obtained in older patients, both in men and in women (Mahaffey and Mergler 1998). By contrast, the median values of Hg concentrations in femoral neck and head samples, determined in patients from two age groups (<60 and >60 years of age) by Lanocha et al. (2012), showed no differences. Miculescu et al. (2011) determined higher concentrations of mercury in the >65 age group in the same type of samples taken from men. Michalak et al. (2014) reported an increase in Hg content of hair samples with age (examined age groups: from 0 to 10 years old to >60 years old). Skalnaya et al. (2016) obtained significantly higher content of Hg in hair samples in patients over 30 years of age compare to younger patients (age group 10–29 years old). Also, the study by McDowell et al. (2004) showed an increase in mercury content in hair with age (examined age groups: from 1 to 5 years old to 16–49 years old). Oppositely, Barbosa et al. (2001) found no statistically significant differences in mercury content in hair samples in tested age group (children and adults). McDowell et al. (2004) has confirmed that total hair Hg is associated with age. The same observations were made by Dumon et al. (1998). Despite this, the accurate mechanism of age-related Hg accumulation in human is not yet fully recognized. One of the proposed mechanisms is linked to impaired excretion of mercury, for which the main way of excretion is its secretion into the gastrointestinal tract and urine. Taking to consideration that aging is linked with reduction of liver and renal functions, the dysfunctions of these organs may lead to damaged mercury excretion (Skalnaya et al. 2014, 2016).

In the group of patients included in the present study, there was no statistically significant difference between the content of mercury in femoral bones and the fish consumption. It should be pointed out that the consumption of fish and seafood is the most common factor that is given as one of the main sources of mercury for humans (Agusa et al. 2007, Mahaffey et al. 2009). In the Asian population, the Hg content of the bones was much higher (2.75 mg/kg dw Hg) than in this study, which was connected with different dietary patterns (people in Asia eat large amounts of fish and seafood) (Chan Yoo et al. 2002). Lanocha et al. (2013) observed higher contents of mercury in the femoral bones of Polish patients who consumed fish and seafood several times a month compared with the patients who did not consume fish and seafood or consume such products only once a month. Also, several other studies confirmed that the frequency of fish and seafood consumption is closely related to mercury content of human tissues (Johnsson et al. 2004; Fang et al. 2012).

Regarding smoking habits and mercury content of the femoral bone, we did not find any associations being in agreement with the observation by Michalak et al. (2014), where the group who declared smoking cigarettes was characterized by lower hair levels of Hg than the non-smoker group.

In this study, we observed higher content of mercury in female patients taking cardiac medications than in those who were not taking such drugs. Kowalski and Frankowski (2015) determined mercury in anticoagulants for ischemic heart disease (B) and in protection against heart and blood vessel disease (M) medications. The obtained contents of mercury were 2.6–15.7 ng/g (with mean 9.0 ± 4.6 ng/g) for (B) and 1.5–476.1 ng/g (with mean 58.9 ± 156.5 ng/g) for (M) pharmaceuticals. It is worthy to underline that in the actual legislation of the European Union, there are no restrictions of mercury level in medicines. Also, the studies by Salonen et al. (1995, 2000) have confirmed the relationship between the accumulation of mercury in the human body and the fast advancement of atherosclerosis. Moreover, they observed 2-fold risk of intense myocardial infarction (AMI) and fatality from coronary heart disease (CHD) and cardiovascular disease (CVD) in male patients with an raised hair content of mercury (>2 μg/g) (Salonen et al. 2000; Salonen et al. 1995). Similar findings about associations between blood mercury level and hypertension and between blood mercury level and myocardial infarction were found in the other studies (Kim et al. 2014; Valera et al. 2013; Tinkov et al. 2015). According to proven relationship between the content of mercury in the human body and cardiovascular disease, the observed higher content of Hg in patients taking medicines associated with this disease may be indicative that the drugs could be an additional source of this metal for human.

It is worth to underline that in the present study, the Hg content of the femoral bone was positively correlated with the BMI, body weight, and width of the femoral bone, which was particularly pronounced in women (Fig. 4). It should be underlined that the correlation between the mercury content of bones and the body mass index (BMI) was not recognized in previous studies. Cho et al. (2014) found positive associations between the BMI and the whole blood Hg levels. In turn, in the Korean study, mercury concentrations in the blood were significantly higher in males than in females. Therefore, Hg concentrations in the blood were associated with low-density lipoprotein (LDL) cholesterol, high-density lipoprotein (HDL) cholesterol, and BMI (You et al. 2011). In contrast, in the Brazilian study, the Hg content of hair was not correlated with BMI (Barbosa et al. 2001). Kim et al. (2010) showed a significant correlation between the waist–hip ratio (but not BMI) and blood Hg concentrations. The Russian study conducted by Skalnaya and Demidov (2007) demonstrated that the obesity in women significantly corresponded to elevated hair Hg content. In other studies, Skalnaya et al. (2014) found the significant correlation between BMI values and hair mercury content both in men and in women. In the Polish study by Michalak et al. (2014), the Hg content of hair positively correlated with BMI. The mechanisms of the relationship between mercury content and body weight are not fully understood (Cho et al. 2014). One of the hypotheses is connected with the influence of the endocrine system (mainly estrogen) on fat tissue and bones (Dermience et al. 2015), which may indirectly confirm the finding that concentrations of Hg in the cortical bone in patients without osteoporosis were about 60 % higher than in patients with osteoporosis (Lanocha et al. 2013). Furthermore, high Hg levels in the blood were linked with a lower risk of osteoporosis in postmenopausal women (Cho et al. 2012). Another probable mechanism of increased metal content in the organism during obesity can be impaired metal excretion, for mercury is biliary secretion (Skalnaya et al. 2014). Besides, Park et al. (2009) found that high mercury concentration in hair may indicate increased risk of metabolic syndrome, which is strongly associated with obesity (Tinkov et al. 2015). This finding is consistent with the statement by Barbosa et al. (2001), that factors, such as gender, age, and BMI, can modulate metabolism of nutritive and toxic metals. Also, the excellent review by Tinkov et al. (2015) presented the role of mercury in pathogenesis of metabolic syndrome components: dyslipidemia, hypertension, insulin resistance and obesity. However, to recognize the full mechanism of the relationship between obesity and Hg body burden, more data of content of mercury in serum, plasma, and also the analysis of enzymes and hormones are needed.

The femoral bones of humans, which were the subject of the present study, are the type of research material which is rarely analyzed for mercury content. The regulations concerning the daily intake of mercury for humans are various, for example, 0.5 μg/Hg/kg/day by Food and Drug Administration (USA) and 0.3 μg/Hg/kg/day by the Agency for Toxic Substances and Disease Registry (USA) (Clarkson 2002).

Yamamoto and Shima (2009) estimated the threshold body burden (TBB) and threshold daily intake (TDI) of human 0.46 mg/kg and 0.0046 mg Hg/kg/day, respectively. In comparison to our results, the obtained concentrations of mercury in femoral bone are lower and they do not pose a direct a threat to the human. It should be emphasized that despite this, it does not change the fact that femoral bone, as well as the hair or blood, can be used as the biological indicator medium for mercury in humans.

## Conclusions

In this study, total mercury content was obtained in all samples of the head and neck of the femur, both in women and in men. In all the examined patients, higher concentrations of mercury were measured in the neck of the femur than in the femoral head. The analysis of the impact of various factors on the mercury content of the femoral head and neck has led to the following conclusions. In all patients, no statistically significant correlations between the Hg content of the FN/FH and the age of patients were found; however, the increase in mercury content of the femoral neck with the age was observed in women, and higher content of mercury in younger patients (<60 years of age) and the oldest ones occurred in men. Based on the results, we supposed that taking the heart disease medicines can affect the accumulation process of mercury in bone tissues of the femur. Also, this hypothesis needs to be confirmed in additional population studies, which will allow to find the association between the accumulation of mercury in bone and types of taking medication. No statistically significant associations between the content of mercury of the femoral bone and the fish consumption, cigarette smoking, drinking alcohol, working with chemical substances, severity of osteoarthritis, pain intensity, and radiological parameters were found. A statistically significant increase in the mercury content of the femoral head and neck with the increase in body weight was obtained. A statistically significant increase in the Hg content of the femoral neck with the increase in body weight, and BMI was statistically confirmed only in women. It is worthy to stress that these findings may be a good source of data of the mercury content in women with different body mass index. A statistically significant correlation between the mercury content and the width of the femoral bone was observed in all patients, while a statistically significant correlation between the mercury content of the femoral neck and the width of the femoral bone was demonstrated only in men. The obtained results point out to the role of bone tissues of the head and neck of the femur in the evaluation of possible human exposure to mercury. To sum up, the toxic effects of mercury are still not fully recognized. Therefore, it is important to find a new indicator of real content of this metal in the human body, especially as, like Clarkson (2002) said, this metal still is considered as element of mystery.
